# Exploring biomedical and traditional care pathways for people with psychosis in Karachi, Pakistan

**DOI:** 10.3389/fpsyt.2023.1086910

**Published:** 2023-07-20

**Authors:** Zahra Khan, Onaiza Qureshi, Aneeta Pasha, Osama Majid, Saniya Saleem, Pasco Fearon, Madiha Shaikh

**Affiliations:** ^1^Interactive Research and Development, Karachi, Pakistan; ^2^Department of Psychology, University of Cambridge, Cambridge, United Kingdom; ^3^Research Department of Clinical, Educational and Health Psychology, University College London, London, United Kingdom

**Keywords:** psychosis, stigma, mental health, traditional healing, treatment pathways

## Abstract

**Background:**

Psychosis is known to have an adverse impact on an individual’s quality of life, social and occupational functioning. A lack of treatment options for psychotic disorders such as schizophrenia contributes to adverse outcomes for individuals. A significant proportion of people with psychosis consult both formal and traditional routes of care. This warrants a need to explore perceptions around treatment options provided by diverse care providers, as the identification of avenues for support can improve psychiatric, alternative treatment and social outcomes.

**Methods:**

Focus groups discussions (FGDs) and in-depth interviews (IDIs) were used. Interactive Research and Development (IRD) research staff conducted 20 IDIs and 2 FGDs to obtain information about the perspectives, treatment pathways and experiences of individuals with psychosis, their caregivers, and service providers. Questions for clinician care providers and faith healers revolved around perceptions of psychosis, service users’ background, subject knowledge and treatment, feedback and referral mechanisms, and promotion of services. A thematic analysis was used to analyze the interviews and coding was conducted on NVivo.

**Results:**

The results were categorized into five themes: perception of psychosis, experience of seeking/receiving care, assessment and diagnosis methods, promotion of services, and living with psychosis. Across service providers and patients, there was a wide variety of causes attributed to psychosis, and an overall lack of awareness regarding severe mental health conditions from both formal and informal care-providers. Biomedical treatment received mixed reviews, while some reported it as beneficial, the limited number of institutes and clinicians to cater for patients, stigma within society and care providers, the burden of caregiving, and misinformation from faith healers were all significant barriers to treatment.

**Conclusion:**

The results highlight the use of traditional healing practices for psychosis in Pakistan, which, coupled with inadequate referral mechanisms, present an opportunity to bridge the treatment gap between clinical and traditional healing practices through integration of treatment within community structures and systems. Better awareness of psychosis and its treatment methods, alongside interventions that reduce stigma could help facilitate help-seeking behavior and reduce the burden of caregiving.

## Introduction

The prevalence of psychotic disorders, including schizophrenia, bipolar disorder, depression with psychotic features, and substance-induced psychoses, and their adverse impact on quality of life is a critical public health issue. Schizophrenia affects about 21 million people worldwide and is globally among the top 20 contributors to years lived with disability, which can be attributed to factors including early adulthood onset and the need for lifelong treatment (1). Moreover, schizophrenia and affective psychosis have a mortality rate that is 2 to 3 times higher than the general population and subsequently people with psychotic disorders face a 15-year reduction in life expectancy (2, 3). The disorder is both debilitating in its symptoms, and due to its early onset, and protracted treatment course, also a financial burden on patients, their friends, family, and the healthcare system overall (1).

Within Pakistan, 1–2% of the population is estimated to be affected by schizophrenia, although the exact number is unknown (4). The estimated duration of untreated psychosis is between about 5 months to over 34 months (5). Obstacles to treatment initiation and success can be understood through the myriad of factors that hamper provision of mental health services in the region. With a population of about 200 million, about 22 million are estimated to have a mental health condition, yet there are only around 500 qualified practicing psychiatrists in Pakistan, which results in a ratio of 1 psychiatrist for 400,000 people (6). There is also a lack of psychiatric and specialty clinics for diagnosis and treatment of psychosis, compounding problems of treatment access. As a result, many people with psychosis in developing countries receive little to no formal care (4).

In addition to the dearth of treatment options for psychosis, a host of social factors in Pakistan contribute to the development and exacerbation of such disorders such as high rate of emotional expression in the home, attribution of symptoms based on cultural misconceptions (e.g., black magic, religious reasons) and poor social support (7). A shortage of alternative housing and community based mental health services means that the burden of care falls on the family members with whom the majority of patients in Pakistan with psychosis reside. There are also limited resources to support these caregivers and families (8).

Moreover, illiteracy, particularly in rural areas where most of the Pakistani population resides, a general lack of awareness of psychosis and the belief in supernatural causes for such disorders result in a significant proportion of people in Pakistan seeking help from faith healers (1). Faith healing is a belief that religion or religious practices can treat certain ailments through the use of prayer or other rituals (e.g., exorcism, physical extraction of disease objects, countering the magical spell, amulets, chanted rings, holy ash, prayers etc.) (9, 10). The efficacy of faith healing in South Asia is debated, while some argue in its favor due to the opportunities for referrals between faith healers and clinicians as shown in African countries (11, 12) and the placebo effect it may offer for mental illnesses such as depression (13, 14). Other perspectives point out the potential harm, either directly through practices such as involuntary restraint, fractures, wounds, or social barriers, e.g., financial burden on marginalized groups and limited autonomy of people with mental health conditions on the treatments (14, 15). The nature and availability of medical treatment may also cause treatment delays. While the exact numbers of the population seeking faith healers for treatment is unavailable, a hospital-based cross-sectional survey in Karachi revealed that about 32% of the psychiatric patients in that hospital had visited a faith healer in their lifetime (9). This help-seeking preference for faith healers is a key factor identified for the under-detection of, and delays in treatment for psychotic conditions. In a Pakistani study it was noted that 61% of all delayed treatment cases were due to limited awareness and knowledge about treatment options for psychosis (16).

For those that pursue clinical or psychiatric services as a treatment option, available mental healthcare for schizophrenia in Pakistan is largely biomedical, i.e., using medications (antipsychotic drugs) and electro-convulsive therapy. Medical and religious healing therapies are either pursued simultaneously, or religious healing replaces psychiatric treatment altogether (1).

Many people with psychosis consult either psychiatrists or faith healers, and sometimes both in conjunction, and otherwise rely heavily on their informal caregivers. Therefore, there is a need to explore the current treatment options utilized by both formal and informal care providers, along with any current or potential intersections in treatment, knowledge, attitudes and practices around psychosis overall. Doing so can provide insight into how barriers to both treatment pathways can be improved, and how different treatment pathways by care providers could complement each other. The aim of this study is to explore referral mechanisms of care providers, types of support available, and obstacles to treatment as perceived by both care providers and people with lived experience of psychosis.

## Methods

### Research questions

1. What is the lived experience of people living with psychosis and their caregivers in Pakistan in terms of type of services available to them, access to services, and quality of services?
2. What are the views of clinical and non-clinical service providers about identification of psychosis and the treatment pathways in Pakistan?

### Study design and context

This study was approved by the ethics committee at UCL (23291/001) and IRD IRB (2022_07_001) in 2021. This was an inductive qualitative study with focus groups discussions and semi-structured interviews utilized as the primary data collection methods. IRD research and field staff members trained in conducting qualitative interviews (OM, MB, and IA) conducted these interviews with the aim of exploring perspectives and experiences of treatment pathways of caregivers and people with lived experience of psychosis, and both clinical and non-clinical (faith/traditional healers) service mental health providers.

### Sample size

Purposive sampling was used to recruit 10 individuals with lived experience of psychosis, 10 care-givers, five clinical service providers, and five non-clinical service providers. The former was identified as an appropriate sample size to reach sufficient data saturation and serve the requirements of the objectives and research design utilized in this study, i.e., “a broad of range of experiences” (17).

Individuals with lived experience of psychosis and caregivers for the study were recruited through outpatient mental health service organizations. Both were approached through clinician referral in specialist facilities which included the Jinnah Postgraduate Medical Centre (JPMC) Department of Psychiatry, Karwan-e-Hayat, House of Pebbles, and Pakistan Association of Mental Health. As each of these institutes consists of service users of varied income class levels, we aimed to recruit an overall sample that is representative of the total population of people with severe mental health conditions in Karachi. Clinical service providers were approached through the same services. Non-traditional service providers such as faith healers were identified and approached through existing networks of the IRD’s Community Engagement Centre in Karachi that serves socio-economically disadvantaged populations in Karachi’s Korangi district.

All 30 participants were over the age of 18 and those that were unable to provide written informed consent or speak in either English or Urdu were excluded. Service users with a prior clinical diagnosis of psychosis (at least 9 months) and who had been receiving treatment for the past 1 year were recruited. The diagnosis of psychosis was defined by the International Classification of Diseases Version 10 (ICD-10) classification of Schizophrenia, schizotypal, delusional and other non-mood psychotic disorders (F20-29) and/or bipolar disorder with psychotic features (F31.2, F31.5, F31.64). Those with dementia and/or significant cognitive impairment and/or severe learning disability, organic psychosis or drug-induced psychosis as the primary diagnosis were excluded. The eligibility and exclusion criteria was shared with mental health clinicians who referred patients based on patient history. Research shows that people access traditional healing before receiving clinical care (4) so by keeping an inclusion criteria of 1 year for receiving treatment, we believe the group will be able to provide perceptions of their help-seeking journey for both bio-medical approaches and traditional healing practices. The inclusion criteria for service users having a diagnosis of psychosis for at least 9 months also ensures that participants have been on appropriate treatment long enough to manage their positive symptoms and will have the capacity to provide consent for research participation. Criteria for caregivers included family members living with or providing supportive care for a relative with psychosis or hired carers (e.g., private 24-h nursing support) providing supportive care for an individual with psychosis for at least 2 months. Clinicians working as a mental health or healthcare professional (including lay workers) and non-clinical service providers with previous or current experience in providing support for individuals with psychosis in a non-clinical setting, were also enrolled. Service providers both clinical and non-clinical without regular contact with individual(s) with psychosis were excluded. Tables 1–4 provide a breakdown of the study populations’ socio demographic characteristics by each stakeholder group.

**Table 1:** Socio demographic characteristics of the study population of faith healers in Karachi’s Korangi district 2022.

Variable	Characteristics	Frequency (*N* = 5)
Age	18–29	3	>40	2
Sex	Male	5	Female	0
Occupation	Imam	2	Paster	2	Other	1
Mother tongue	Saraiki	1	Punjabi	4

**Table 2:** Socio demographic characteristics of the study population of caregivers in Karachi’s Korangi district 2022.

Variable	Characteristics	Frequency (*N* = 10)
Age	18–29	1	30–39	1	40–50	3	>50	2
Sex	Male	7	Female	1
Occupation	Employed	3	Self-employed	1	Unemployed	1	Housewife	1	Retired	1
Mother tongue	Urdu	4	Punjabi	2	Balti	1

**Table 3:** Socio demographic characteristics of people with psychosis in Karachi’s Korangi district 2022.

Variable	Characteristics	Frequency (*N* = 10)
Age	18–29	3	40–50	2
Sex	Male	5	Female	5
Occupation	Employed	1	Self-employed	1	Unemployed	4
Mother tongue	Urdu	4	Bulti	1	Memon	1

**Table 4:** Socio demographic characteristics of clinical care providers in Karachi’s Korangi district 2022.

Variable	Characteristics	Frequency (*N* = 5)
Age	18–29	1	30–39	3	>40	1
Sex	Male	3	Female	2
Occupation	Psychiatrist	3	Community Psychiatrist	2
Mother tongue	Urdu	4	Balochi/Sindhi	1

### Data collection procedures

Semi structured interviews for 10 people with psychosis, five clinicians, five faith healers, and two Focus Group Discussions (FDGs) with a mixed group of five caregivers each were conducted face to face. Caregivers expressed an interest in a group discussion and it was felt that the collective experience of caregiving will normalize their experiences as caregivers share common traits and experiences. The interviews and discussions were conducted by a trained male researcher (OM) employed by IRD, with relevant educational qualifications, and who was trained in qualitative methods by (OQ) the program manager at IRD, and (MS) a lecturer and researcher at UCL, and was assisted by two IRD field coordinators (MB, IA). The interviews took place in 2021, at the research office at IRD in Karachi or online via Zoom. The interviews were conducted in Urdu based on participant preference and were audio-recorded to be transcribed and translated from Urdu to English, prior to the data analysis. No repeat interviews were conducted. The average length of interviews ranged from around 11–55 min (29 min on average) for service providers, 10–33 min (16 min on average) for people with lived experience of psychosis, while the two focus group discussions with care-givers lasted between 55 and 84 min. We found that patients preferred to finish their interviews sooner as they took place after their routine appointments and they may have had limited attention for the subsequent engagement. A semi-structured interview guide was prepared in Urdu based on an existing topic guide that was developed through consensus discussions with clinical and public health researchers from Pakistan, India, and the United Kingdom for a situational analysis on psychosis treatment protocols (18) within Karachi. The topic guide was amended for the current study through consultations within the research team to ensure consistency and relevancy for the research aims.

The interview schedule included general knowledge around psychosis, experiences living with psychosis and accessing and utilizing services for people with psychosis and caregivers. Questions for clinician care providers and faith healers revolved around patient background, subject knowledge and treatment, feedback and referral mechanisms and promotion of mental health services. Revisions to the interview guide included (1) exploring the journey of someone seeking care for psychosis; recognition of own condition, duration of symptoms before seeking care, types of entry points for care, i.e., formal/informal, experiences and the barriers and facilitators of care seeking through different types of formal and informal care, what the service user understood to be “recovery” or “wellbeing” within the scope of their condition.

(2) Level of understanding that carers have of their relative’s condition, duration of symptoms before seeking care, types of entry points for care for their relatives, i.e., formal/informal, experiences of caregiving for relatives with psychosis, and the barriers and facilitators of care seeking through different types of formal and informal care.

(3) Exploring how formal (clinical) and informal (tradition/faith healers) care providers understand psychosis, how they assess it in their populations, what kind of symptoms they see in practice, what kind of treatment/approaches they use to reduce symptoms or manage the condition, what stage patients come to them, the feedback and referral mechanism they use for their services, how they promote their services and how often they follow-up with their population.

### Data analysis

Data analysis for IDIs and FGDs were conducted using an inductive approach where themes were identified based upon repeated and important topics for discussion in the interviews rather than on pre-existing theoretical concepts. An inductive experiential framework was the position from which the current data were analyzed. This assumes that the data gives access to individuals’ meanings, lives and realities accurately. Interpretation of the data is therefore grounded in the assumption that the interview process has allowed access to participants’ real experiences. A Critical Realist approach was assumed, that is, that the data allows access to an existing reality/ truth about stakeholders experience of psychosis and its treatment, and that this data provides useful knowledge about how to implement and make recommendations from these practices (19). Interview transcriptions were proof-read for identifying information, and then analyzed using principles of Thematic Analysis by Braun and Clarke (20). The first two stages of analysis included familiarization and preliminary coding by authors OM and OQ, after which a final codebook was developed and iterated by ZK through open coding. This allowed for calibration which we explored informally through differences in coding between OQ, OM and ZK. Interviews were subsequently entered into QSR NVivo and final coding was conducted by authors ZK, MS and OQ. The next four stages included searching for themes, reviewing themes with the wider research team via consultation, (reviewed across four consultations), defining and naming themes and then articulating findings for the report. Given the limited resources for the research, we were not able to contact participants to provide feedback on the findings.

## Results

Overarching themes discussed in this paper include perception of mental health and psychosis, assessment and diagnosis, experiences of providing or receiving support, living with psychosis and promotion of services. Sub-themes for each stakeholder group are presented in Supplementary material 1.

### Perception of mental health and psychosis

For people with psychosis their understanding of psychosis ranged from the notion that treatment should be provided for it, that it does not improve on its own, while another patient believed there is no hope for a cure or improvement, and one claimed it was caused by God’s will. There was also a wide array of causes attributed to its onset, including suddenly feeling sick or running away, while others reported stress from negative thinking, domestic issues, increasing cost (such as rent). Caregivers also reported some kind of inciting incident or trauma (e.g., robbery, death, departure of a loved one) as reasons for onset.

Interestingly, individuals with psychosis and caregivers did not seem to explicitly attribute psychosis to supernatural causes, however they did initiate seeking support from faith healers.

Clinician perception of the various manifestations of psychosis were dependent on causal attributes; substance abuse, genetics and/or chemical imbalances were identified as the most prominent causes that lead to the development of psychosis. Types of familial upbringing were also considered a contributor to the development of psychosis symptoms, while situational factors such as imprisonment or irregular daily routines were also thought to be responsible. One clinician also went on to assign personal responsibility in the development of psychosis and claimed that it was dependent on one’s own coping skills.

Faith healers identified three broad spiritual reasons for the development of psychosis which included (1) possession by black magic or evil spirits or satanic influences, (2) a lack of faith/God’s will or (3) worries about domestic issues. Specific conditions for spirit possession include going to isolated or dirty places, engaging in black magic, or committing sins against God.

Accordingly, prayer or spiritual healing was seen as the most integral, if not the only component for healing/improving the patient’s condition by four faith healers. However, two faith healers also did report some recognition of a distinction between spiritual possession and psychological issues as causes, with the former requiring faith healing and the latter requiring medication. In this case, one faith healer suggested that prayer can be an initial outlet for psychological relief, but if “satan is playing with their mind” (Faith Healer_04) then medical care is required.

*“So, if it is an issue with spirits, that is not something that can be fixed using medication, it is necessary that there should be qur’anic recitation or through amulets, but patients with psychological problems can also be treated using scripture but generally their issue is resolved using medication or medical purpose” (Faith Healer_01)*

### Assessment and diagnosis

Clinicians conducted assessments through symptoms described or exhibited by people with psychosis and history taking. Notably, the most popular approach for diagnosis was through family reports of history and symptoms, and this was regarded as the most valuable mode of information. This was linked to the justification that patients did not have sufficient insight into their own condition for accurate self-reporting. “A lot of these patients do not have basic insight so we have to inquire through their relatives.” (Clinician_01) One clinician reported making a judgment on how health literate patients are based on their socioeconomic status, and sharing bio-medical information accordingly.

*“When a patient does come to the clinic we normally examine them based on the health-belief model. We assess their economic status and also look closely at their literacy level. If an educated person comes and I tell them the symptoms, they will do a Google search and all and their queries will be accordingly.” (Clinician_02)*

Faith healers did not report any one predominant way of diagnosing patients. Assessment is done through intuition, examining/ asking about the effects of the condition, behavior, and certain key tells such as patients that scream and make noise are known to be ones that have an evil spirit possession. One faith healer reported a similar sentiment to clinicians where people with psychosis were seen as lacking autonomy, or were not acknowledged as independent individuals.

“*First is that you have to talk to them with kindness it can happen anytime that the person who is feeling this we don’t trust them for example if a child tells me that I’m feeling this or that then the child is told immediately to sit down and that they don’t know anything. The child is feeling this so they are telling us we need to trust them and listen and then investigate what the child has said about the problem.” (Faith Healer_05)*

### Experiences of providing or receiving support services

Caregivers described a lack of support available for psychosis overall. One participant noted that rural areas do not have institutes equipped to deal with the treatment of psychosis, and while cities have institutes to treat mental health illnesses, overall, the participants provided mixed reviews regarding their effectiveness. Two caregivers also reported a lack of good quality doctors, hospitals or treatment options, and some mentioned that even the doctors/ psychiatrists available do not have the time capacity to treat all their patients. “Despite paying multiple visits for 4–5 days we could not approach the said doctor, so we left this option as it was a waste of time.” (Caregiver Focus Group Discussion 1).

*“As I told you earlier about the institute in Hyderabad where I kept my brother. But they asked us to take our patient because either he would kill himself or any other person as he kept fighting. I think the people there were not satisfied with their services.” (Caregiver Focus Group Discussion 1).*

Conversely, bio-medical treatment, specifically medications, was also reported to have a positive impact on patient symptoms by three caregivers in focus group discussion one, who detailed how medications prescribed by doctors provided effective treatment outcomes, two of which described the effectiveness of treatments that were precluded by testing and/or a longer diagnosis time.

*“[Hospital name] staff is very nice, they welcomed us very nicely, I have taken medicines from them for my brother and it has improved him a lot.” (Caregiver Focus Group Discussion 1).*

People with psychosis had mixed opinions about the benefits of available treatment. Regarding medication, three reported adverse effects such as nausea, facial swelling, a ‘bad reaction’, or simply lack of efficacy in improving symptoms. Five patients claimed that medical treatment led to at least some improvement, with one reporting that s/he experienced a relapse shortly after discontinuation. For four people with psychosis, overall treatment options took more trial and error to get right; medications were either switched, or their dosage tapered to reduce side effects, while three others switched doctors and/or institutes before finding suitable ones. Obstacles in seeking care included high costs of travel and medication, long wait times at the hospital and medication cycles, which essentially means that if a patient misses the day of medication distribution, they have to wait for the next cycle to acquire it at the respective institution (every 15 days according to two patient reports).

Electroconvulsive therapy was deemed ineffective by caregivers in both focus group discussions, with one participant reporting that its traumatic effects lasted for years.

The duration of untreated psychosis ranged from 1 to 3 years. Social stigma of mental health conditions in Pakistani society was flagged as a major obstacle in seeking out treatment, and the lack of awareness about psychosis, especially among families is a major issue that prompts certain clinicians to provide psychoeducation to caregivers and patients as part of their treatment plan.

Another barrier to treatment as reported by three clinicians, includes patients going to faith healers rather than clinicians for their treatment, which, as reported by one clinician, delays treatment. “The problem here is that patients usually come here after knocking several doors and speaking to Pirs [A holy man or faith healer] believing that their condition might have something to do with Jinns [spirits]. In one of my workplaces, it is so common for patients to visit us after 8–10 years of illness.” (Clinician_03). One clinician also stated that faith healers sometimes provide the wrong information about psychosis.

People with psychosis echoed similar sentiments regarding the ineffectiveness of Faith healing as a treatment for psychosis. Three people with psychosis reported that faith healers whose treatments included the use of amulets and holy water, were unable to successfully treat their psychosis.

*“These were babas (faith healers) in [name of rural setting], they used to make some amulets and give it to us. But these were of no use to us.” (psychosis_09)*

*“Participant: He gave me holy water (water upon which he recited quran).*

*Interviewer: So, that did not work for you?*

*No, it didn't.” (psychosis_08)*

One person with psychosis also stated that he/she had been possessed by a spirit and that spiritual healing characterized by reciting ‘surahs’ had reduced the feeling of such a possession, although it is unclear who the person (referred to as “someone told me to do this”, that recommended this was, and whether or not they were a faith healer (Person with lived experience_06).

Faith healers reported approaches that predominantly involved prayer, provision of amulets, and recitation of holy texts which based on their own reports sometimes also led to the improvement of symptoms. Spirit possession, which was most reported as the cause for psychosis symptoms, is also detected and removed/treated through prayer, recitation of holy texts, use of cleansing oils and the person’s own strength of faith. Four faith healers reported positive results in patients’ conditions following the use of either prayer, amulets, or rituals around elimination of spirits. “When we do pray for them they get better as a result of God’s mercy (Faith Healer_04).”

However, one faith healer also detailed relapses in patients’ conditions, or instances where patients screamed in pain or were burned with oil. When these approaches do not work, responsibility is attributed to the patient’s own strength of faith, or the evil spirit. One faith healer also reported using medication to treat patients for such an instance.

*“Our usual method is such that whenever we find someone if they have a one time issue, then we give them taweez and other treatment items together. We tell them that if they start feeling better after 40 days, they should definitely come and tell us about it. If we find however that their issue is longer lasting then we give them the medication for 11 or so days. When they return after 11 days, we check them and see whether they have reported any improvements.”_(Faith Healer_02)*

Clinicians reported that the cost of care can be high and certain organizations provide free of cost medication/treatment. In our sample, medication was provided by four clinicians and the dosage, time of administration and type varied based on the stage and symptomatology of psychosis. Three clinicians also reported providing counseling and psychoeducation and claimed that it helps with medical adherence.

Caregivers also shared the perceived burden of caregiving which was reported as a negative experience by family members with psychosis. One caregiver reported that their own mental health is affected during the process of providing support to people with psychosis, and that they have to suppress their own needs for the sake of the patient.

An overall lack of psychoeducation provided by service providers, and a subsequent lack of awareness among people with psychosis and their caregivers is reflected in the findings around seeking support and perceptions of causes. The types of information provided to caregivers also play a role (both negative and positive) in accessing support. Two caregivers reported that faith healers directed them toward doctors, out of which one claimed that they had wasted a lot of time going to faith healers based on others’ recommendations.

### Promotion of services

Clinicians primarily gained service users through referrals by other clinicians and/or facilities (such as outpatient departments of hospitals), or references by relatives or service users. Psychiatrists that practice for a long time also reportedly attract service users based on their reputation or popularity.

*“So I talk about myself, it has only been 2-3 years since I have started practicing. So either my colleagues refer patients to me, or I get them through this hospital, or even my immediate circle refer patients to me. Dr.[name] is very reputed in this field. Patients mostly come here from various areas of Pakistan (Quetta, Peshawar, Gilgit) to have consultations with him. (Clinician_3)”*

Three clinicians also reported different methods of self-promotion including setting up mental health camps, conducting awareness sessions on psychosis, the use of billboards, distributing pamphlets in the community, or following up with registered service users in the area.

*“We go into the community one day before camp and the next day set camp, if the camp is decided on Monday so we go into the community on Friday for ground activities. And for the registered patients of that area we have their contact number and full data, we call them prior for their follow up session.”(Clinician_05)*

Like clinicians, faith healers also reported that most visits by service users occurred due to references by others, and usually from people that were previously treated. Two faith healers also discussed a demand for spiritual treatment, specifically by ‘God-fearing’ individuals, and there was a perception that generally people in the faith healers’ communities gravitate toward religious leaders and/or treatments.

*“In our community people have this awareness that if there is something wrong with us then we need to go to God’s house.” (Faith Healer_2)*

Two faith healers also mentioned utilizing self-promotion techniques, such as either the use of social media platforms or announcements in mosques about specific services offered.

### Living with psychosis

People with psychosis reported that living with psychosis included internal psychosocial experiences such as restlessness, feeling hyper, being worried and stressed out “I used to feel worried or anxious.”(Person with psychosis_1), experiencing delusions “I felt as if I am in Heaven” (Person with psychosis_6), and fixating on certain past events.

“*I start remembering really old things, stuff like x person said this to me, y person did this to me. I started remembering old thoughts.” (Person with psychosis_7)*.

There was also a general perception or hope among participants that treatment and/or medication would lead to an improvement in psychosis symptoms. “All I thought at that moment was that dad is giving me medication, I will get better.” (Person with psychosis_2).

The most common physical sensations that accompanied psychosis were reportedly headaches or some type of mental strain. “Those symptoms were that I would feel heavy headedness with stress on my mind, I used to get severe headaches and yes that was it.” (Person with psychosis_2) Less common symptoms discussed included the feeling of burning, experiencing seizures or ‘jerks’, insomnia, breaking things, running away, feeling hyper, forgetfulness, dizziness, and one participant also reported a loss of consciousness.

*“Afterwards, when I felt angry beyond measure, my heartbeat skyrocketed. After that people used to tell me oh you had a seizure, when i regained consciousness I regained control of my muscles, After 20-25 minutes of this, I started realizing my name, who I am, who I am not, then slowly and steadily I started remembering who I was.” (Person with psychosis_9)*

## Discussion

### Key findings

The exploration of lived experiences described in this study include an account of what it is like to live with psychosis in Karachi, the various types of services available and utilized by different care providers, and barriers to treatment including stigma, lack of awareness, lack of treatment options, and attitudes of care-providers. Components that appeared to be the most consequential for treatment pathways, include implications of clinical and faith healing practices on treatment outcomes and the role of caregivers in aiding some clinicians’ diagnoses, and a subsequent need for their psychoeducation and support services. ‘Perception of mental health and psychosis’ and ‘experiences of seeking or receiving care’ themes cut across all stakeholders groups. However, clinicians, faith healers and people with psychosis differed in their ‘perception of psychosis’, particularly regarding the attributable causes. Clinicians focused on biological and psychosocial aspects of psychosis, people with psychosis characterized their illness through causal life events, and unsurprisingly faith healers provided spiritual explanations. Interestingly, individuals with psychosis and caregivers did not explicitly attribute psychosis to supernatural causes often, however they did initiate contact with faith healers. Experiences of providing care also varied based on participant group in terms of types of support offered, with caregivers offering emotional and day-to day support, faith healers providing spiritual care in the form of prayers and amulets, and clinicians utilizing medication or counseling as their primary mode of treatment. People with psychosis were roughly split between whether they found medication useful, while more caregivers reported it as beneficial than those who did not.

### Strengths and limitations

The diversity of relevant stakeholders interviewed in our study deepened our understanding of complex issues around psychosis and provided multiple perspectives. Doing so also allowed us to corroborate narratives around the experiences of barriers to treatment and identified the need for collaborative approaches between clinical and non-clinical providers to caring and treating people with psychosis. Limitations of this research are that the recruitment of study participants was done primarily through large-scale mental health clinical sites in Karachi, and so the sample size may not be representative of the larger population of people with psychosis, especially those belonging to illiterate groups. Participants were interviewed after their routine appointments to reduce any additional transportation burden to take part in the interviews, however this may have led to limited capacity for attention and as a result interview durations from service users were shorter than clinicians and caregivers. There is also missing socio-demographic data for seven of the 30 interviewed participants (mainly caregivers and people with psychosis) due to time constraints and being unable to collate this information via a follow up call.

### Comparison of findings and possible interventions supported by global literature

#### Implications of clinical and faith healing practices on treatment outcomes

There are various factors that contribute to inaccessibility, delays, or ineffectiveness of treatment overall, and these are based on both the quantity and quality of treatment and availability of service providers. Regarding clinician treatment specifically, patient satisfaction with medication was at times positive although quite a few did report adverse symptoms, and, more importantly some caregivers stated that doctors lacked time and were difficult to seek out for treatment. Clinicians echo back similar sentiments, with one participant claiming that they limit the number of new patients recruited as those ones require more time initially. This is unsurprising, as Pakistan has a severe shortage of psychiatrists and mental health experts overall, and WHO estimates less than one psychiatrist is available for every 100,000 people in most of South-East Asia (21, 22). While clinician availability is one aspect of the delay in treatment, our study also highlights another possible deterrent from timely and appropriate medical treatment; pursuing faith healers.

With the onset of psychosis largely attributed to supernatural causes by faith healers, it can be inferred that people with psychosis and caregivers who go to faith healers are not informed of biopsychosocial causes and treatments. Moreover, our results showed that faith healer treatment practices can be characterized as quite extreme and risky, such as using oil to eliminate spirits and service users being burnt or subjected to pain. Another country where faith healing is highly prevalent is India, and a study conducted in rural Gujarat reflects some overlaps with our findings regarding the methods employed in faith healing, including the use of prayer, amulets, and patients ingesting smoke or the use of fire (23). This further emphasizes the scale and consistency of such potentially harmful practices within faith healing treatment overall.

Delays reported in acquiring treatment due to time spent visiting faith healers is a crucial finding in our study (and is at times, explicitly stated by clinicians and caregivers). This finding is important because rapid access and initiation of appropriate treatments for psychosis is critical, and meta-analyses and a longitudinal study on first episode psychosis show that a prolonged duration of untreated psychosis is associated with both a reduction in treatment response, as well as “symptomatic and functional recovery” in first episode psychosis (24, 25).

Moreover, faith healing practices also vary in perception of effectiveness and severity. Faith healers’ decision making and rationale for referral to medical treatment is also unclear, further highlighting the need for collaborative thinking between clinicians and psychoeducation to help identify and treat psychosis effectively. However, this is not to diminish the value of spirituality in recovery from mental health conditions entirely as spirituality and religious beliefs have also been shown to be beneficial to mental health in global and Asian literature (26, 27).

It is also worth mentioning that while repercussions have been reported for faith healers replacing the role of psychiatrists (28), there have been multiple historical and current accounts of abuses experienced by patients within psychiatric facilities as well (29). It can be tempting to implicate a certain provider with inhumane care, patients will always be at risk of abuse without effective legal regulations and structural provisions to protect the rights of people with psychosocial disabilities in those settings (30).

The two caregiver reports in our study, of faith healers referring them to clinicians, provide an example of how treatment integration through the creation of referral pathways between faith healers and clinicians, could reduce delays in treatment. A related intervention is worth exploring how faith healing is a common practice, but most faith healers do not seem aware or trained in biopsychosocial practices that could be leveraged to provide more effective treatment for psychosis.

Such studies both emphasize the need for holistic care, and a better understanding of psycho-spiritual approaches for it, while considering the potential harm and opportunity costs of not accessing care from other sources. This need is punctuated by the limited availability of clinical service providers in the country. Coupled with the findings from our study which highlight the prevalence, pitfalls and opportunities of directing service users to appropriate treatment plans around faith healing, this leads to the notion that perhaps it would be beneficial for both clinical and traditional service providers to be trained in, and aware of the other’s treatment practices, for the purpose of reducing treatment delays and improving the overall quality, and availability of care.

#### Stigma

Our study underscores the various levels and types of stigmas that people with psychosis may face, even at times, from service providers themselves. Results highlighted that clinicians have beliefs about the onset and causes of psychosis which place undue responsibility on families and people with psychosis themselves, for example blaming upbringing as well as people’s capacity to manage stress. Although psychosocial stressors are a widely accepted risk factor for psychosis (31), they are often not isolated and rather psychosis can be conceptualized as a combination of biopsychosocial stressors. Similarly, faith healers’ beliefs that one’s spirituality and strength of faith is both a contributing factor to psychosis, but also an important consideration in its treatment, further punctuates the finding that service providers have some internalized stigma when treating people with psychosis. Conversely, clinicians report stigma as a major obstacle that delays people accessing treatment. Caregivers and people with psychosis share similar views regarding stigma and its consequences, including familial isolation and ostracizing neighborhood environments.

#### The unique burden of caregiving for psychosis and the need for caregiver specific support

Another crucial component of care provision for psychosis in Pakistan is caregivers. Our results outlined the significant role played by caregivers in the diagnosis and clinical treatment of people with psychosis. Most notably, feedback on symptoms and treatment outcomes by caregivers is often sought and relied upon more by clinicians than that offered by the patients.

Informal caregiving for people with psychosis has a unique burden associated with it, most notably giving rise to mental health issues in the caregiver, and requiring a large amount of time spent caregiving (32). Caregivers in our study detailed giving up one’s own desires/needs for the relative with psychosis, experiences of stigma from other family members and community members as previously discussed, and the considerable emotional and mental strain of caring for someone with psychosis. Caregiving is universally reported as posing a significant burden and resulting in mental and physical health consequences. A study using GHQ-28 as a measure of distress among caregivers of people with first episode psychosis revealed a notable amount of distress and caregiving burden (33). Studies in India and Malaysia report that caregivers of people with psychosis face significant levels of emotional distress. In Malaysia, about 31.5% of caregivers who participated reported mild to great psychological distress which was “found to be associated with community rejection” (34). Similarly in India, caregivers for people with schizophrenia also attributed perceived reduction in social support along with stigma and stress to their emotional distress in providing care (35). Globally, a study conducted in five sites across the US has reported similar findings; caregivers for people with treatment resistant schizophrenia found providing emotional care particularly taxing and reported that both their family and romantic relationships have been negatively impacted by caregiving. Physical health impacts including insomnia, headaches, pain, and depression were also reported (36). While our study did not reveal any adverse physical effects for caregivers, these are worth exploring in the future.

More importantly, these findings highlight the need for caregiver support interventions. A randomized controlled trial conducted with primary caregivers of patients with schizophrenia in Turkey found that psychoeducation and tele-psychiatric follow ups reduced caregiving burden, depression and high expressed emotions (37). An RCT in Pakistan evaluated the feasibility of delivering a Culturally adapted Family Intervention (CulFI) based on a Pakistani derived conceptual framework and had promising results with high participation and retention percentages of 90% (38). The demonstrated feasibility of developing psychosocial care pathways for people with psychosis and their families and or caregivers, warrants further trials and implementation for relieving the burden of caregiving reported in our study.

### Implications of the findings for future research

These findings reflect the importance of exploring ways to support caregivers of people with psychosis in Pakistan who appear to be facing similar challenges of stigma and social isolation seen in global trends. The findings highlight the varied understanding and explanations for psychosis and its symptoms among all groups but especially for people with psychosis trying to make sense of their experiences. Subsequently, our findings also highlight the importance of collaborative working between clinical and non-clinical providers to shorten the duration of untreated psychosis, identify and treat psychosis utilizing a holistic approach informed by a biopsychosocial and spiritual framework. The impact of bridging referral gaps between clinical and faith-healing practitioners is yet to be explored in a Pakistani context.

Timely psychoeducation, raising awareness schemes and anti-stigma campaigns can be beneficial in helping to demystify unhelpful explanations as well as providing a culturally sensitive understanding of psychosis that is informed by an inclusive biopsychosocial-spiritual approach. Integration of psychoeducation and awareness particularly in community settings, and where faith healing is predominant, should be explored.

Developing culturally relevant psychological interventions to deal with emotional distress caused by Pakistan-specific stigma and misinformation (such as a lack of faith leading to the development of psychosis), could be a potential area for relieving caregiver burden and improving treatment outcomes for people living with psychosis, and warrant future research.

### Conclusion

The results emphasize the significance and prevalence of traditional healing practices for psychosis in Pakistan and underscore the need to address the gaps and potential for integrating traditional and clinical practices for increased access and acceptability of mental health therapeutic approaches. Further exploration into supporting caregivers of people with psychosis, and the specific need for raising awareness in Pakistan to counter stigma and social isolation shows promise and has been punctuated by the study results.

## Data availability statement

The raw data supporting the conclusions of this article will be made available by the authors, without undue reservation.

## Ethics statement

The studies involving human participants were reviewed and approved by Interactive Research and Development IRD_IRB_2022_07_001 University College London, UCL Ethics Project ID Number: 23291/001. The patients/participants provided their written informed consent to participate in this study.

## Author contributions

OQ, MS, and OM conducted protocol development. OM carried out data collection. OQ, MS, and ZK contributed to the data analysis and manuscript development. AP, SS, and PF assisted with edits and a final review of the manuscript. All authors contributed to the article and approved the submitted version.

## Funding

This study was funded by University College London’s, Global Innovation Fund and Psychology and Language Sciences Departmental funds. Funds for the open access publication fee was shared between UCL and IRD Pakistan.

## Conflict of interest

The authors declare that the research was conducted in the absence of any commercial or financial relationships that could be construed as a potential conflict of interest.

## Publisher’s note

All claims expressed in this article are solely those of the authors and do not necessarily represent those of their affiliated organizations, or those of the publisher, the editors and the reviewers. Any product that may be evaluated in this article, or claim that may be made by its manufacturer, is not guaranteed or endorsed by the publisher.
