# Supplementary material for: Exploring biomedical and traditional care pathways for people with psychosis in Karachi, Pakistan
Source: Front Psychiatry. 2023 Jul 20;14:1086910. doi: 10.3389/fpsyt.2023.1086910 (PMC10411590; doi:10.3389/fpsyt.2023.1086910)
Supplement: Supplementary file 1 [file Data_Sheet_1.docx]

Appendix 1

Interview Guide for Participants

**Questions for people who have psychosis (IDIs)**

***General Knowledge around psychosis***

1. What information do you have around psychosis or psychotic disorders (prompt: symptoms of psychosis, cause of psychosis)
   1. What is the term you use for people with this condition?
2. What do you think is the treatment or support that should be provided to people seeking care for this condition? (prompt: beliefs around traditional/alternative approaches or biomedical treatment offered in facilities)

***Experiences wrt living with psychosis***

1. Could you describe the first time you experienced symptoms? What were the symptoms?
2. How long after the presentation of your first symptoms did you receive a diagnosis for psychosis?
3. What has been your experience after having been diagnosed with this condition?
   1. What does the diagnosis mean to you?
   2. What has your personal experience been like after being diagnosed with these symptoms? (prompt: change in quality of personal relationships, socialising, work life, daily responsibilities
4. What has been your experience with pursuing treatment for psychosis? (prompt: barriers, difficulties, accessibility of healthcare systems or alternative support systems)
5. What does recovery look like for you? / What does wellbeing mean to you?

***Experiences with accessing and utilizing services***

1. Do you go to access services for your condition?
2. How do you go about accessing services for your condition (potential probes: logistical support, transportation, availability of medications, accessibility to formal psychiatric services)?
3. Where do you go to access services?
4. What has been your experience of accessing services?

#

#

#

# Questions for families of people who have psychosis (FGDs)

***General knowledge around psychosis***

1. What information do you have around psychosis or psychotic disorders (prompt: symptoms of psychosis, cause of psychosis)
   1. What is the term you use for people with this condition?
2. Do you have any knowledge of different services/approaches to treatment of psychosis?
3. What services/approaches would you prefer when receiving treatment for your condition?
4. When did you first observe the presentation of symptoms of psychosis in your relative?
5. What kind of behaviors did you see in your relative after the onset of their condition?
6. What has been your experience in providing care/support to your relative? (potential probes if not discussed: social, economic, mental and emotional, lack of support networks)
7. Do you accompany your relative to access services for their condition?

***Experiences with accessing and utilizing services***

1. How long did it take for your relative to receive a formal diagnosis of psychosis and when were they able to access help
2. What are the types of help you sought or received for your relative’s condition?
3. How do you go about accessing services for your relative’s condition (potential probes: logistical support, transportation, availability of medications, accessibility to formal psychiatric services)?
4. Where do you go to access services?
5. What has been your experience of accessing services for your relative?

#

#

#

# Questions for clinical care providers (including general practitioners and mental health professionals) (IDIs)

***Background of the patient***

1. What is the socio-economic or demographic background of the patients that come to you with psychosis?(Gender,age)

***Subject Knowledge and Treatment Methods***

1. What information do you have around psychosis or psychotic disorders (prompt: symptoms of psychosis, cause of psychosis)
2. What do you think is the treatment or support that should be provided to people seeking care for psychosis? (prompt: beliefs around traditional/alternative approaches or biomedical treatment offered in facilities)
3. What are the main methods you use for the treatment and support of patients with psychosis?
4. At what stage do patients with psychosis usually present to you and with what symptoms? (probe: duration of untreated psychosis)
5. What sort of training is available to you for the treatment and management of patients with psychosis?

***Feedback and Referral Mechanism***

1. What is the general feedback you receive from your patients in terms of the treatment you provide.(prompt: treatment, improvements in condition)
2. Are people with psychosis referred to you? (what sort of people are referred to you, who refers people to you?)
3. How often are other patients with psychosis referred to you?
4. What is the normal duration of follow-up consultations you offer to patients with psychosis?

***Promotion of services***

1. How do you promote your services to people with psychosis (prompt: in the facility and the community?

**Questions for traditional and faith healers (including imams, pirs/fakirs etc) (IDIs)**

1. What is the socio-economic or demographic background of the people that come to you with this condition? (Prompt: gender/age)

***Subject Knowledge and Treatment Methods***

1. What is the term you use for people seeking care for this condition?
2. What information do you have around psychosis or psychotic disorders (prompt: symptoms of psychosis, cause of psychosis)
3. What do you think is the treatment or support that should be provided to people seeking care for this condition? (prompt: beliefs around traditional/alternative approaches or biomedical treatment offered in facilities)
   1. What are the main methods you use for treatment and support of patients with this condition?
4. At what stage do people with this condition usually present to you and with what symptoms?
5. What sort of techniques/approaches do you utilize in dealing with people with this condition? (prompt: magic/craft/scriptures)

***Feedback and Referral Mechanism***

1. What is the general feedback you receive from these patients in terms of the treatment you provide.(prompt: treatment, improvements in condition)
2. Are people with this condition referred to you? (prompt: how often do you see people with this condition in your practice, who is the party that refers these people to you?
3. What is the normal duration of follow-up check ups that you offer to patients with psychosis?

***Promotion of services***

1. How do you promote your services to people with this condition? (prompt: how do people with this condition know where to access your services?)

Appendix 2


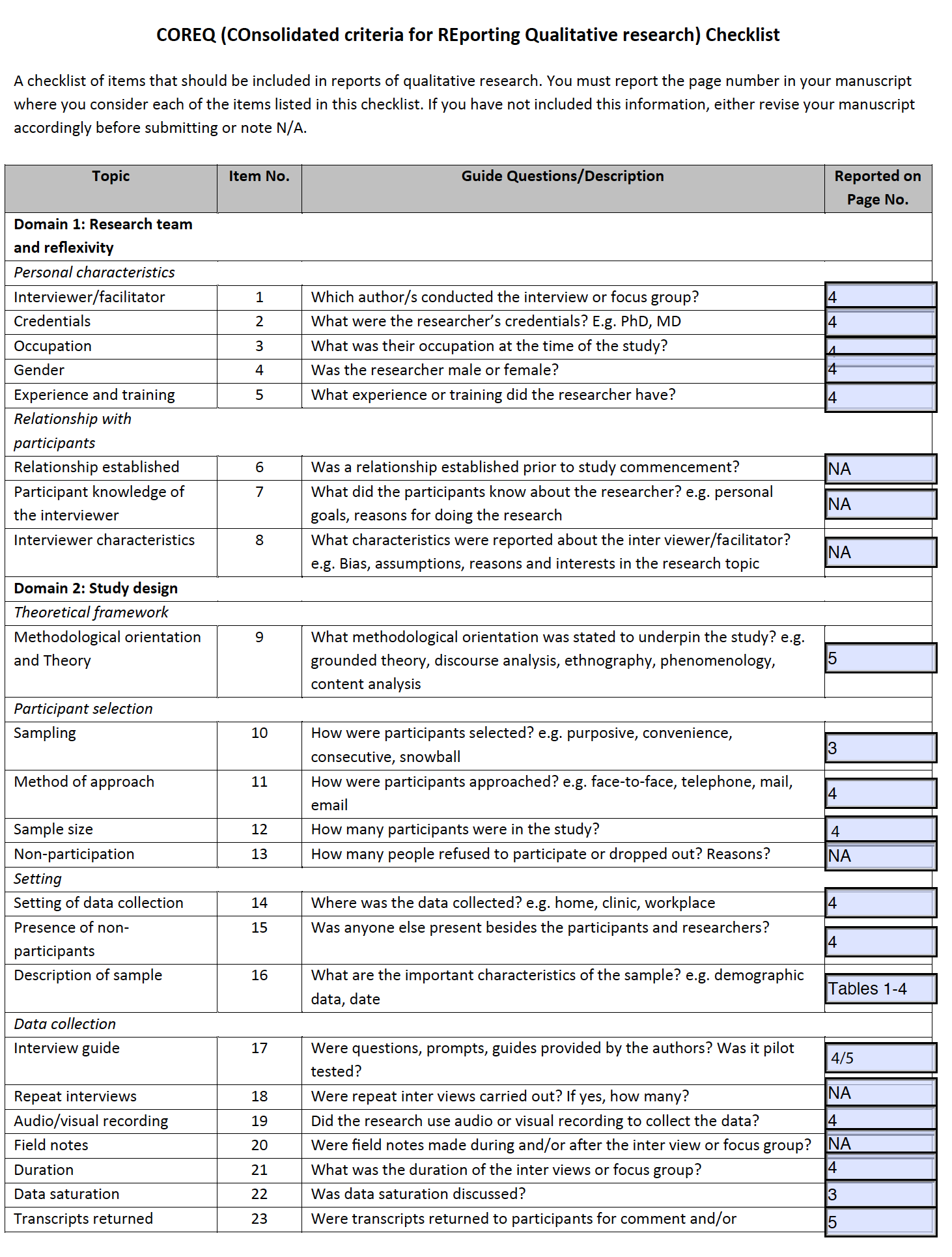


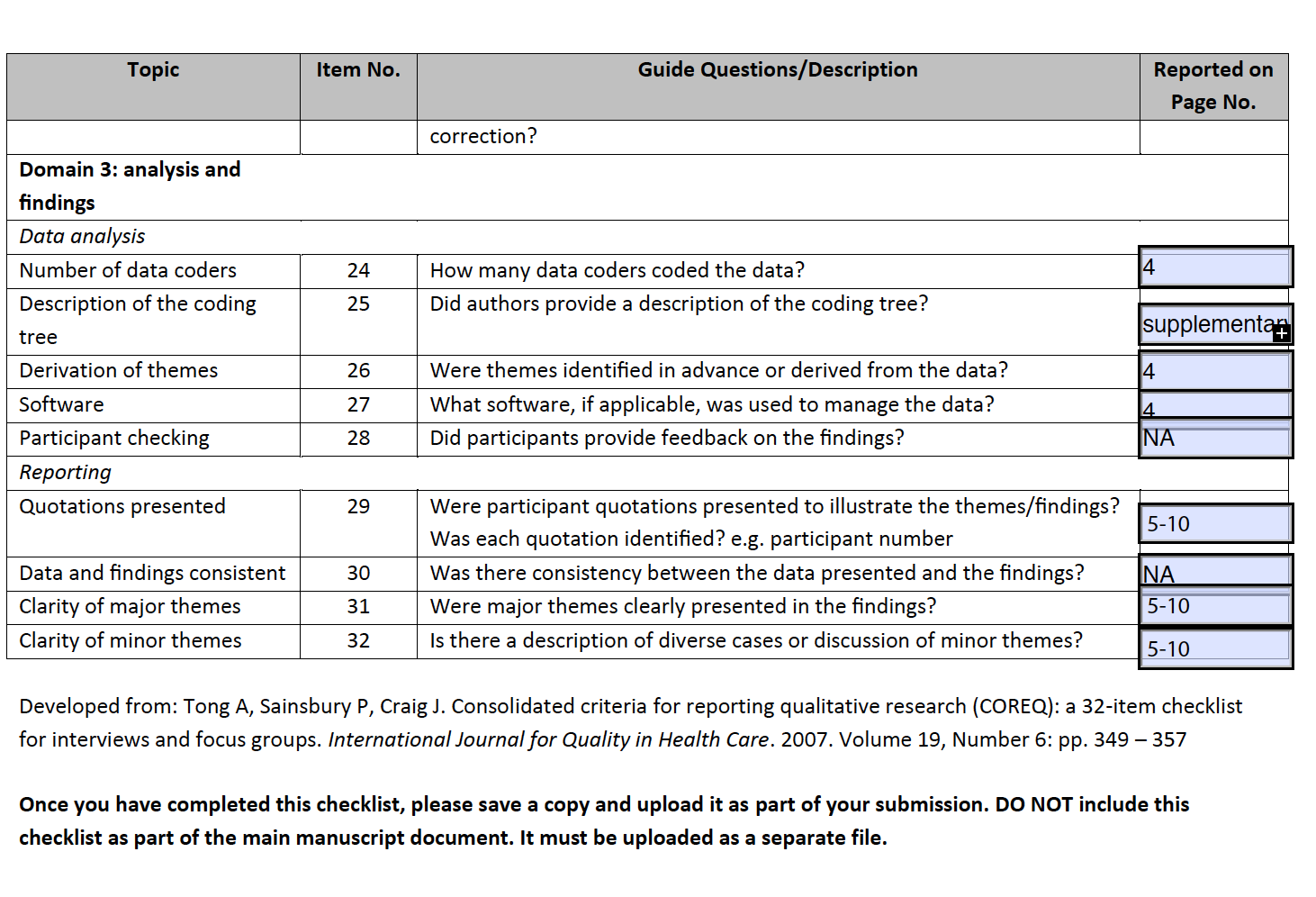


Supplementary material 1

| **Theme** | | **Group** | **Sub-theme** | | ***Quotations*** |
| --- | --- | --- | --- | --- | --- |
| Perception of Mental Health & Psychosis | | FH, C | Population Served | | *"We usually have lower socioeconomic class and lower middle income class patients." _C_02* |
|  |  |  |  |  | *"It is a combination of patients, we get a combination of normal and affluent patients."_FH_02* |
|  |  | CG, P, FH | Causes/ Onset | | *“So parenting styles are a major cause of the development of this condition…In other settings, such as in prison when prisoners are tortured, they develop psychotic symptoms and the other main cause is substance abuse which includes “powder”/Heroin, LSD and ICE and ‘Crystal’ /Meth.” -C_01* |
|  |  |  |  |  | *"Okay, so often what happens is that people genuinely are facing issues with spirits, if they have gone to an isolated or spooky place and have defecated there or played around there, that is also a popular cause of this issue." - FH_01* |
|  |  | FH | Spiritual Healing | | *"So, if it is an issue with spirits that is not something that can be fixed using medication, it is necessary that there should be quranic recitation or through amulets, but patients with psychological problems can also be treated using scripture but generally their issue is resolved using medication or medical purposes"* |
|  |  |  | Manifestation of mental health conditions | | *"For them, lots of times what happens is such people have to be tied down at home and there are many that face these issues periodically. In normal circumstances, they will be fine  but suddenly they will start getting violent and start speaking gibberish".-FH_01* |
|  |  | C | Bio-medical Information | | *" Then for purely psychotic spectrum disorders like acute psychotic disorder, schizophrenia, schizophreniform, schizoaffective form (they) are all psychotic spectrum and start with psychotic features. Then it's substance use or organic brain syndrome like delirium then in that we can find (psychosis)."_C 02* |
|  |  |  | Socio-cultural perspectives | | *"According to me there should be more research related to mental health especially with regards to practices or management protocols there is a room for improvement in this area."_C 04* |
|  |  | CG | Symptoms | | *"Usually in psychosis the patients start showing irritation. He doesn’t share true things with family members. If any guest comes into the house, then he talks to them in a loud voice, misbehaves, makes mockery of them, or talks nonsense."_CG 01* |
|  |  |  | Duration | | *"He has been suffering from this disease for almost 18 years. He also got admitted to the hospital more than 3 to 4 times._CG 02"* |
|  |  | P | How people with psychosis act | | *"Then they start getting hyper and start becoming seriously mentally ill"_P06* |
| Assesment/ Diagnosis Methods | | C | Procedures used | | *"Yes so we take the conversation further and try to pick up on questions based on things that the patient is discussing. If a patient reports that they believe everyone is against them, we would ask about what makes them think like that. We are able to get some idea about the thought process of a patient this way."_C02* |
|  |  |  |  |  | *"We have a process of interview so during the time of interview we get the idea the patient has come up with this type of psychosis."_C04* |
|  |  | FH | Approaches taken | | *"Yes, mostly through discussion you can understand. If we have a walk-in patient who tells us that someone has done black magic on them. We try to notice their symptoms/their behavior and then make an assumption about their condition."_FH02* |
| Promotion of services | | C, FH | Methods of promotion | | *"So as I mentioned, when people get better, they refer the service to other people. That is how it works."_FH04* |
|  |  |  |  |  | *"We work in a community, we distribute our pamphlets to different places in that community like barber shops, beauty parlors, maternity homes, private clinics of General Physicians and we request them to refer these patients to us if they encounter any. We treat them free of cost, and what happens in some family they have locked in such patients when they see pamphlet they brought them to our OPD"_C05* |
|  |  | FH | Demand for services | | *"Yes, people find their way to us themselves. When we start doing these things, you will find people coming to you themselves "_FH02* |
| Experiences providing or receiving support | | All groups | Types of support offered/ accessed | | *"Usually, we give them a taweez, it can be in amulet form or holy water form."_FH01* |
|  |  |  |  |  | *"I think doctors are very busy nowadays. They don’t have time to talk to the family members because the number of patients has increased and we don’t have specialized doctors."_CG02* |
|  |  |  | Frequency | | *"I come here after 15 days for a checkup and to get medication."_P08* |
|  |  |  | Availability | | *"Yes, in 19 most of the patients were for follow-ups so we check their prognosis, often patients come for prescrItion refills. But if I talk about per day, around 18 to 20 is the average of patients."_C_02* |
|  |  | C, FH | Feedback mechanism | | *"…Usually we take feedback from caregivers because the patient of psychosis never tells us exactly about themselves." _C_05* |
|  |  | FH | Perception of patients experience | | *"They feel at ease thinking they are feeling healthy or that they will feel better soon."_FH 03* |
|  |  |  | Experience of care provider | | *"People share such graphic details about their life that we fall into deep confusion about what we should do with them or what we should provide for them that gives them hope. Often, we give them things that help them deal with their problems in the best manner and their issue gets resolved also."_FH01* |
|  |  | P | Cost | | *"He said, we will treat you. One of the doctors quoted a price of Rs. 3500 some would say it would cost you Rs. 3000. He said you will get better in a week"_P08* |
|  |  |  | Time taken | | *"No, we normally have to wait for 15-20 minutes but our turn does come"_P01* |
|  |  |  | DUP | | *"Not really, the awareness raising session conducted at [hospital name] was my first introduction to this, I was informed then that I am carrying these symptoms since 6/7 month to a year at least."_P06* |
|  |  | CG | Factors for consideration in seeking support | | *"Honestly it’s all about money. The more you spend, the more facilities you will get. Overall it is an expensive process. Sometimes such patients don’t go to the hospital with their family members, so we have to call someone from the hospital to handle their aggressiveness and take them to the hospital."_CG 02* |
|  |  |  | Impact on family | | *"The neighborhood also remains disturbed and when the children from this house go out in schools etc then people talk about them and taunt them for the illness of their family member. People don’t have an understanding of the treatment etc."_CG 01* |
|  |  |  |  |  | *"We have to give importance to the patient. We have to favor them in whatever they say. The immediate family members like sisters, brothers and parents are attached to the patients and they can make a difference. But sometimes we also become irritated after seeing the patient’s condition.."_CG01* |
| Living with Psychosis | | P | Physical or somatic expression | | *"Body pain. Also when I lost consciousness it was due to weakness."_P04* |
|  |  |  | Internal psycho-social experiences | | *"My head becomes really heavy and my brain feels burdened"_P02* |
|  |  |  | Social, Occupational and Interpersonal experiences | | *"Very simply put, I do not want anything anymore. Neither money, nor marriage, everything is gone. I am only focused on building a future so that I do not become a druggie, I would like it if someone helps me get employment but I will be okay if no one does so."_P06* |
| **Key** | | | |  |  |
| C | Clinician | | |  |  |
| CG | Caregiver | | |  |  |
| FH | Faith Healer | | |  |  |
| P | People with psychosis | | |  |  |
